# Prawn Shell Chitosan Has Anti-Obesogenic Properties, Influencing Both Nutrient Digestibility and Microbial Populations in a Pig Model

**DOI:** 10.1371/journal.pone.0144127

**Published:** 2015-12-04

**Authors:** Áine M. Egan, Torres Sweeney, Maria Hayes, John V. O’Doherty

**Affiliations:** 1 School of Agriculture and Food Science, University College Dublin, Belfield, Dublin, 4, Ireland; 2 School of Veterinary Medicine, University College Dublin, Belfield, Dublin, 4, Ireland; 3 Teagasc Food Research Centre, Ashtown, Dublin, 15, Ireland; INIA, SPAIN

## Abstract

The potential of natural products to prevent obesity have been investigated, with evidence to suggest that chitosan has anti-obesity effects. The current experiment investigated the anti-obesity potential of prawn shell derived chitosan on a range of variables relevant to obesity in a pig model. The two dietary treatment groups included in this 63 day study were: T1) basal diet and T2) basal diet plus 1000 ppm chitosan (*n* = 20 gilts per group (70 ± 0.90 kg). The parameter categories which were assessed included: performance, nutrient digestibility, serum leptin concentrations, nutrient transporter and digestive enzyme gene expression and gut microbial populations. Pigs offered chitosan had reduced feed intake and final body weight (P< 0.001), lower ileal digestibility of dry matter (DM), gross energy (GE) (P< 0.05) and reduced coefficient of apparent total tract digestibility (CATTD) of gross energy and nitrogen (P<0.05) when compared to the basal group. Fatty acid binding protein 2 (*FABP2*) gene expression was down-regulated in pigs offered chitosan (P = 0.05) relative to the basal diet. Serum leptin concentrations increased (P< 0.05) in animals offered the chitosan diet compared to pigs offered the basal diet. Fatness traits, back-fat depth (mm), fat content (kg), were significantly reduced while lean meat (%) was increased (P<0.05) in chitosan supplemented pigs. Pigs offered chitosan had decreased numbers of *Firmicutes* in the colon (P <0.05), and *Lactobacillus* spp. in both the caecum (P <0.05) and colon (P <0.001). *Bifidobacteria* populations were increased in the caecum of animals offered the chitosan diet (P <0.05). In conclusion, these findings suggest that prawn shell chitosan has potent anti-obesity/body weight control effects which are mediated through multiple biological systems *in vivo*.

## Introduction

The potential of natural products for preventing obesity is now being investigated, due to synthetic compounds having some harmful side effects. Chitosan, is a non-toxic, natural polysaccharide composed of randomly distributed β-(1–4)-linked d-glucosamine and *N*-acetyl-d-glucosamine and has been shown to control body weight [1, 2]. The control of body weight is influenced primarily by three interrelated factors; food intake, nutrient absorption and body fat stores [3]. Studies have shown that dietary supplementation of chitosan reduces feed intake in both mice [4] and pigs [2] and has been associated with an increase in serum leptin levels. Leptin, a hormone produced by adipocytes has been shown to play a key role in appetite suppression [5]. While the anti-obesity effects of chitosan are thought to originate from its lipid-binding properties [6] other mechanisms are also worthy of exploration. Chitosan alters gut microbial populations as evidenced by measurable effects on odour emissions in pigs [7]. Hence other anti-obesity effects of chitosan may be mediated through the modulation of gut microbiota [7, 8] and subsequent alterations to nutrient absorption which favour a leaner body weight.

Reports that obesity is associated with alterations to the gut microbiota suggest a potential connection between specific microbial taxa and this disorder [9]. The predominant phyla in the large intestine (*Firmicutes*, *Bacteroidetes* and *Actinobacteria)* have an important role in the fermentation of non-digestible carbohydrates which are broken down into short chain fatty acids, the quantities of which are linked to obesity [10].

Nutrient digestibility is an important determinant in identifying the volume of nutrients absorbed by a host. Walsh *et al*. [2] demonstrated that dietary supplementation of chitosan increased faecal fat excretion, suggesting that there was reduced lipid absorption. The small intestine is the primary site for fat absorption and transport. Following nutrient breakdown by digestive enzymes, nutrient transporters facilitate the absorption of nutrients into the blood stream [11]. At this point, these nutrient transporters act as the gatekeepers of overall nutrient status and subsequent growth and development of the animal [12]. Non-optimal changes to both gene expression and activity of nutrient transporters and/or the gut microbial populations have been implicated in both restricted and excessive growth [12, 13]. Such changes may underpin the reported anti-obesogenic properties of chitosan, subsequently altering nutrient availability.

The objective of the present study was to investigate the effects of dietary supplementation of chitosan on performance, nutrient digestibility, serum leptin concentrations, nutrient transporter and digestive enzyme gene expression and gut microbial populations using a pig model. The hypothesis is that dietary supplementation of chitosan will reduce dietary intake, body weight and carcass fat content with a down-regulation of long chain fatty acid transporter and digestive enzyme gene expressions, while altering gut microbial populations in favour of a leaner body weight.

## Materials and Methods

All experimental procedures described in this experiment were approved under University College Dublin animal research ethics committee (AREC) and conducted under experimental license from the Irish Department of Health in accordance with the Cruelty to Animals Act 1876 and the European Communities (Amendments of the Cruelty to Animals Act, 1876) Regulations (1994).

### Generation of chitosan from chitin

The chitosan was generated from prawn (*Nephrops norvegicus*) shell sourced from Spiddal, Co. Galway, Ireland. Prawn shell was collected on five separate occasions before chitosan extraction was carried out. The prawn shell was heated in boiling sodium chloride (4% NaCl) for 10 min and cooled in tap water to remove excess prawn protein material. The shell was washed extensively and freeze-dried. Clean, dry shell was milled, sieved and subsequently demineralised and deproteinised using a BioFlo 110 Modular Bioreactor (New Brunswick Scientific, USA). Following this, HCL (0.25 M) was added to the prawn shell material at a ratio of 1:40 weight/volume to demineralise the shell. The temperature of the reaction was maintained at 40°C for 6 h. The shell material was subsequently drained, washed until a neutral pH was obtained, then frozen and freeze-dried to obtain a demineralised shell powder. The demineralised shell powder was then deproteinised using 0.25 M NaOH using a shell to solvent ratio of 1:40 w/v at 70°C for 6 h. The chitosan was washed until a neutral pH was reached, then freeze-dried and subsequently milled to obtain chitosan powder.

### Characterisation of chitosan

The molecular weight data for the extracted chitosan was analysed using the SEDFIT-MSTAR. The degree of acetylation was determined by analysis of the 1H proton spectrum following the method of Muzzarelli *et al*. [14].

### Animals and management

The experiment was a complete randomised design. Forty females pigs (Large White x Landrace genetic lines, Hermitage, Co. Kilkenny, Ireland), with average body weight of 70 kg, SD = 0.9, were randomly assigned to one of two dietary treatments (20 animals/treatment): (T1) basal diet (control) and (T2) basal diet plus 1000 ppm chitosan. Female pigs were used because of their higher back fat deposition relative to male pigs [15]. The concentration of chitosan used in the present study was based on previous work by Walsh *et al*. [2]. The diets were provided *ad libitum* in a meal form and water was available *ad libitum* from nipple drinkers. The diets were formulated to have similar digestible energy (14 MJ/kg) and standardised ileal digestible lysine (8.5 g/kg) contents. All amino acid requirements were met relative to lysine [16]. Detailed ingredient composition and chemical analysis of the diets are presented in Table 1.

**Table 1:** Composition and chemical analysis (g/kg, unless otherwise indicated).

Ingredient (g/kg)	T1	T2
Wheat	382.6	382.6
Barley	250.0	250.0
Soya bean meal	170.0	170.0
Maize	150.0	150.0
Soya oil	18.0	18.0
Limestone	12.5	12.5
Salt	5.0	5.0
Monocalcium phosphate	6.6	6.6
Vitamins and minerals premix^a^	2.5	2.5
Lysine HCL	2.3	2.3
L-threonine	0.5	0.5
Chitosan	0	1.0
**Analysis (g/kg, unless otherwise stated)**		
Dry matter	857.6	856.4
Crude protein (N X 6.25)	177.9	177.7
Ash	42.5	42.8
Gross energy (MJ/kg)	15.9	15.7
Neutral detergent fibre ^†^	130.5	130.3
Lysine^†^	9.2	9.1
Methionine and cysteine^†^	5.5	5.4
Threonine^†^	6.2	6.3
Tryptophan^†^	1.9	2.0
Calcium^†^	9.4	9.4
Phosphorous^†^	5.8	5.7

T1, basal diet; T2, basal diet plus 1000 ppm chitosan.

^a^ The premix provided vitamins and minerals (per kg diet) as follows: 4.2 mg of retinol, 0.07 mg of cholecalciferol, 80 mg of α-tocopherol, 120 mg of copper as copper sulphate, 100 mg iron as ferrous sulphate, 100 mg of zinc as zinc oxide, 0.3 mg of selenium as sodium selenite, 25 mg of manganese as manganous oxide, 0.2 mg of iodine as calcium iodate on a calcium sulphate/calcium carbonate carrier, 2 mg of thiamine, 15 μm of cyanocobalamin, 7 mg of pantothenic acid, 2 mg of riboflavin, 7 mg of niacin, 3 mg of adenine and 100 mg of phytase (Natuphos) (Nutec, Co. Kildare, Ireland).

^†^ Calculated for tabulated nutritional composition [19].

The animals were penned in four groups of ten with a space allowance of 0.75m^2^ per pig. The pens were equipped with single space computerised feeders (Mastleistungsprufung MLP-RAP; Schauer Agrotronic AG, Sursee, Switzerland), as described by Varley *et al*. [17] and Walsh *et al*. [2] which allowed individual *ad libitum* feeding and daily recording of dietary intake. Briefly, when the animal entered the feeder, it was recognised by the electronic system (MLP-Manager 1.2; Schauer Maschinenfabrik Ges.m.b.H and CoKG, Prambachkirchen, Austria). Each animal was ear-tagged with a uniquely coded transponder and the identification circuit recorded the number of the animal. When the animal finished feeding and withdrew from the trough, the electronic system recorded the difference between the pre- and post-visit trough weight and the data was stored in a file with the number of identification of the animal, the date, and the time of entry and exit. The animals were weighed at the beginning of the experiment (day 0) and every two weeks up to slaughter (day 63).

Feed samples were collected weekly and stored at -20°C for chemical analysis. Celite (300 mg/kg) was added to the feed at manufacture in order to measure the coefficient of apparent ileal digestibility (CAID) and coefficient of apparent total tract digestibility (CATTD) using the acid insoluble ash technique [18]. Faecal samples were collected daily from each animal during days 28–30 to measure the CATTD using the acid insoluble ash technique.

### Blood sample collection

Blood samples (10 ml) were collected from each animal from the *vena jugularis* by puncture into vacutainers (Becton, Dickinson, Drogheda, Ireland) on day 0 (prior to commencing of the experiment), day 14, 28, 37, 49 and 63 to facilitate leptin quantification. Blood samples were allowed to clot at 4°C and serum was collected after centrifugation (1,500 × g for 15 min at 4°C). Serum samples were stored at -20°C until analysis.

### Post slaughter sample collection

On day 63, all animals were slaughtered after stunning with carbon dioxide and the entire digestive tract was removed by blunt dissection. Digesta samples were recovered aseptically from the ileum in a section approximately 30 cm in length from the ileo-caecal valve, in order to measure the CAID of nutrients. Digesta sample was collected from the caecum and the second loop of the ascending colon, using sterile instruments. Digesta samples were stored at -20°C in separate, sterile containers (Sarstedt) for further volatile fatty acids (VFA) (colon) and microbial (caecum and colon) analysis. Tissue samples from the duodenum (10 cm from the stomach), jejunum (60 cm from the stomach) and ileum (10 cm from the ileo-cecal valve) were collected to analyse the gene expression of nutrient transporters and digestive enzymes. Tissue samples were emptied and cleaned by dissecting along the mesentery and rinsing using sterile PBS (Oxoid) as described previously [20, 21]. Tissue sections of 1 cm^2^, which had been stripped of the overlying smooth muscle were cut from the tissue and stored in RNAlater^™^ solution (Ambion Inc, Austin, TX) overnight at 4°C. The RNAlater^™^ was then removed and the tissue sample was stored at -70°C until RNA extraction.

### Carcass analysis

Backfat thickness was measured at 6 cm from the edge of the split back at the level of the third and fourth last ribs using the Hennessy grading probe (Hennessy and Chong, Auckland, New Zealand). The lean meat content (g/kg) was estimated according to the following formula [2]:

Estimate lean meat content (g/kg) = 543.1 − 7.86x + 2.66y

Where x is fat depth (mm) and y is muscle depth (mm).

Further carcass data were determined using the following equations:

Carcass weight (kg) = hot carcass weight x 0.98.

Kill-out proportion (%) = carcass weight/ body weight (BW).

Estimated ash content = 3% of carcass weight

[22].

Carcass fat content = carcass weight − (lean + ash content of carcass).

## Laboratory analysis

### Leptin quantification

Serum leptin was quantified by using a specific pig leptin enzyme-linked immunosorbent assay (ELISA) kit from Life Science Inc. (Wuhan, China) according to the manufacturer’s instructions. Sensitivity of the assay was 0.114 pg/ml and intra-assay coefficient of variation was < 12%. Absorbance was measured at 450 nm against 570 nm for each assay by using the ELISA plate reader. All samples were assayed in triplicate in the same assay.

### Chemical analyses

Feed, faecal and digesta samples were analysed for nitrogen (N), dry matter (DM), ash, gross energy (GE), neutral detergent fibre (NDF) and oil. The DM content of the feed, faeces and digesta were determined after drying for 72 h at 55°C. The crude ash content of diets, faeces and digesta was determined after ignition of a weighed sample in a muffle furnace (Nabertherm, Bremen, Germany) at 550°C for 6 h. The GE of diets, faeces and digesta samples was determined using an adiabatic bomb calorimeter (Parr Instruments, IL, USA). The N concentration of diets, faeces and digesta was determined using a LECO FP 528 instrument (Leco Instruments, U.K. Ltd, Stockport, Cheshire, UK). The oil content was determined using the Ether Extract Method B [23]. The acid insoluble ash content of feeds, faeces and digesta was determined according to the method of McCarthy *et al*. [18]. Concentration of VFA was determined with minor adaptations as described previously by O’ Connell *et al*. [24].

### Extraction and quantification of microbial DNA from caecum and colon

Microbial genomic DNA was extracted from digesta samples from the caecum and colon using a QIAamp DNA stool kit (Qiagen) in accordance with the manufacturer’s instructions. The quantity and quality of DNA were assessed using a Nanodrop apparatus (ND1000, Thermo Scientific). For the quantitative PCR (QPCR), standard curves were prepared with pooled aliquots of faecal DNA as described previously [25] and used for the absolute quantification of bacteria [26]. Genus- and species- specific primers (Table 2) were used for the estimation of selected bacterial groups based on gene copy number (GCN) in the faecal matter using QPCR on the ABI 7500 QPCR System (Applied Biosystems Limited). For bacterial groups, QPCR was carried out in a final reaction volume of 20 μl containing 1 μl of template DNA, 1μl of forward and reverse primers (100 pM), 10 μl of SYBR Green PCR Master Mix (Applied Biosystems Limited) and 8 μl of nuclease-free water. The thermal cycling conditions involved an initial denaturation step at 95°C for 10 min followed by 40 cycles of 95°C for 15 s and 65°C for 1min. Dissociation analyses of the QPCR products were carried out to confirm the specificity of the resulting QPCR products. All samples were prepared in duplicate. The mean threshold cycle values from the duplicate of each sample were used for calculations. The estimates of GCN for select bacteria were log-transformed, and they are presented as GCN/g digesta.

**Table 2:** Swine-specific primers used for real-time PCR.

Gene	Primer (5' → 3')	Product Length	T_m_ (°C)
*Bacteriodes*	F: AACGCTAGCTACAGGCTT	276	54
	R: CAAATGTGGGGGACCTTC		
*Firmicutes*	F: GGAGYATGTGGTTTAATTCGAAGCA	126	59
	R: AGCTGACGACAACCATGCAC		
*Lactobacillus*	F: TGGATCACCTCCTTTCTAAGGAAT	340	55
	R: TGTTCTCGGTTTCATTATGAAAAAATA		
*Bifidobacteria*	F: GCG TGC TTA ACA CAT GCA AGT C	129	55
	R: CAC CCG TTT CCA GGA GCT ATT		
*Enterobacteria*	F: CATTGACGTTACCCGCAGAAGAAGC	190	58
	R: CTCTACGAGACTCAAGCTTGC		

### Volatile fatty acid analysis

Digesta samples collected from the colon were mixed with sodium benzoate and phenylmethylsulfonyl fluoride to stop any bacterial activity and minimise the effects of post-thawing fermentation which would influence final VFA concentrations. A 1.0 g sample was diluted with distilled water (2.5 × weight of sample) and centrifuged at 1,400 × g for 4 min at 20°C. One millilitre of the subsequent supernatant and 1 mL of internal standard (0.5 g of 3-methyl-n-valeric acid in 1 L of 0.15 mol/L oxalic acid) were mixed with 3 mL of distilled water. After centrifugation to remove the precipitate, the sample was filtered through Whatman 0.45-μm polyethersulfone membrane filters into a chromatographic sample vial. A 1.0 μL quantity was injected into a model 3800 Varian gas chromatograph with a 25 m × 0.53 mm i.d. megabore column (coating CP-Wax 58 (FFAP) CB; Model CP7614, Varian, Middelburg, the Netherlands).

### Nutrient transporter and digestive enzyme gene expression—RNA extraction, complementary DNA synthesis and quantitative PCR

Total RNA was extracted using Trizol^™^ and further purified using the GenElute^™^ Mammalian Total RNA Miniprep Kit (Sigma-Aldrich, Corporation) according to the manufacturer's instructions. Total RNA samples were treated with DNase I (Sigma-Aldrich).

Total RNA was quantified using a NanoDrop-ND1000 spectrophotometer (Thermo Fisher Scientific, Inc.). RNA integrity was assessed on the Agilent 2100 Bioanalyzer version A.02.12 (Agilent Technologies, Inc.) and all RNA integrity number (RIN) values were > 8.9. Complementary DNA (cDNA) was synthesised from 1 μg of total RNA using the Superscript^™^ III First-Strand Synthesis Kit (Thermo Scientific) and oligo (dt) primers following the manufacturer's instructions. The final reaction volume of 20 μl was then adjusted to 120 μl using nuclease-free water. The quantitative PCR (QPCR) assay mixtures were prepared in a total volume of 20 μl, containing 10 μl Fast SYBR PCR Master Mix (Applied Biosystems, Foster City, CA, USA), 1.8 μl forward and reverse primer mix (300 nm), 5.7 μl nuclease-free water and 2.5 μl cDNA. The QPCR was carried out in duplicate on the 7500 ABI Prism Sequence Detection System (Applied Biosystems, Foster City, CA, USA). Thermocycling conditions were as follows: 95°C for 10 min for one cycle, followed by 95°C for 15 s and 60°C for 1 min for forty cycles. Dissociation analyses of the QPCR products confirmed the specificity of all targets. All primers for the selected nutrient transporters: glucose transporter 1 (*GLUT1/ SLC2A1)*, *GLUT2/ SLC2A2*, *GLUT5*, *GLUT7*, sodium-glucose linked transporter 1 (*SGLT1/ SLC5A1)*, fatty acid binding protein 2 *(FABP2/I-FABP2)*, cluster of differentiation 36 (*CD36/FAT*) and peptide transporter 1 (*PEPT1/ SLC15A1*), and digestive enzymes: lipase and maltase are presented in Table 3. All primers were designed using the Primer Express^™^ Software (Applied Biosystems, Foster City, CA, USA) and synthesised by MWG Biotech (Milton Keynes, Buckinghamshire, UK). All samples were prepared in duplicate. The mean cycle threshold values of duplicates of each sample were used for calculations. The optimal number of reference targets were identified using the geNorm application within the qbase PLUS software package (Biogazelle, Zwijnaarde, Belgium). Briefly, the geNorm algorithm on the qbase+ package (Biogazelle, Gent, Belgium) calculated the expression stability factor (M). From this the optimal combination of reference genes required for normalisation were selected. Using this algorithm, reference genes are ranked based on their M values. In brief, geNorm calculates the stability measure M for a reference gene as the average pairwise variation (V) for that gene with all other tested reference genes. A Vn/n+1 value is calculated for every comparison between two consecutive numbers (n and n+1) of candidate reference genes. Following the stepwise exclusion of the least stable reference genes, by the geNorm program, M values were re-calculated and the stability series obtained. Finally, the NF was calculated, as the geometric mean of the most stable reference genes, and the normalised relative quantity (NRQ) of the target genes obtained as the ratio between the relative quantities and the sample specific NF. The basic formula for relative quantification (RQ = 2^ddCt) assumes 100% amplification efficiency (E = 2). The most stable housekeeping genes for the duodenal, jejunal and ileal tissues were: peptidylprolyl isomerase A (*PPIA*) and hydroxymethylbilane synthase (HMBS).

**Table 3:** Swine-specific primers used for real-time PCR.

Gene	Accession no.	Primer (5' → 3')	Product Length	Efficiency %
*SGLT1*	NM_001164021.1	F; GGCTGGACGAAGTATGGTGT		
		R; ACAACCACCCAAATCAGAGC	153	90
*PEPT1*	NM_214347.1	F; GGATAGCCTGTACCCCAAGCT		
		R; CATCCTCCACGTGCTTCTTGA	73	98
*GLUT1*	XM_003482115.1	F; TGCTCATCAACCGCAATGA		
		R; GTTCCGCGCAGCTTCTTC	61	100.90
*GLUT2*	AF054835.1	F: CCAGGCCCCATCCCCTGGTT		
		R: GCGGGTCCAGTTGCTGAATGC	96	107
*GLUT5*	EU012359	F: CCCAGGAGCCGGTCAAG		
		R: TCAGCGTCGCCAAAGCA	60	143
*GLUT7*	XM_003127552.3	F: ACATCGCCGGACATTCCATA		
		R: GCGAGGACTGCAGGAAGATC	75	106
*FABP2*	NM_001031780.1	F: TCGGGATGAAATGGTCCAGACT		
		R: TGTGTTCTGGGCTGTGCTCCA	102	98
*CD36*	NM_001044622.1	F:GGAGAAAAGATCACTACCATCATGAG		
		R: CTCCTGAAGTGCAATGTACTGACA	78	91.6

F, forward; R, reverse; *SGLT*, sodium-glucose linked transporter; *PEPT*, peptide transporter; *GLUT*, glucose transporter; *FABP*, fatty acid-binding protein; *CD36*, cluster of differentiation 36.

### Statistical analysis

The growth performance was analysed by repeated measures analysis using the PROC MIXED procedure of SAS [27]. The model used included pen and animal within pen as random effects. The fixed effects were: treatment, time and interaction between treatment and time. The data on carcass characteristics was analysed using the PROC MIXED procedure of SAS [27]. The model used included pen and animal within pen as random effects. The fixed effect was treatment. Initial body weight was used as a covariate for growth performance data. The leptin data was analysed by repeated measures analysis using the PROC MIXED procedure of SAS [27]. The model used included the pig as a random effect. The fixed effects were: treatment, time of sampling and the associated two way interaction between treatment and time of sampling. The data on nutrient digestibilities, microbiology and VFA’s were analysed using the general linear model procedure of the Statistical Analysis Systems Institute [28]. The model used included the effect of treatment. Gene copy numbers of selected bacteria were log-transformed before statistical analysis. The statistical model used for the nutrient transporter gene expression data analysis included small intestinal region (duodenum vs. jejunum vs. ileum), chitosan inclusion and interaction between region and chitosan inclusion, followed by Bonferroni's test. The probability level that denotes significance is P< 0.05, while P values between 0.05 and 0.1 are considered numerical tendencies. Data are presented as least-square means with their standard errors of the means.

## Results

### Characterisation of prawn shell chitosan

The degree of acetylation obtained was 15%. The average molecular weight of the prawn shell chitosan was 124,000 ± 10,000 g/mol.

### Dietary intake, body weight and carcass characteristics

**Fig 1:**
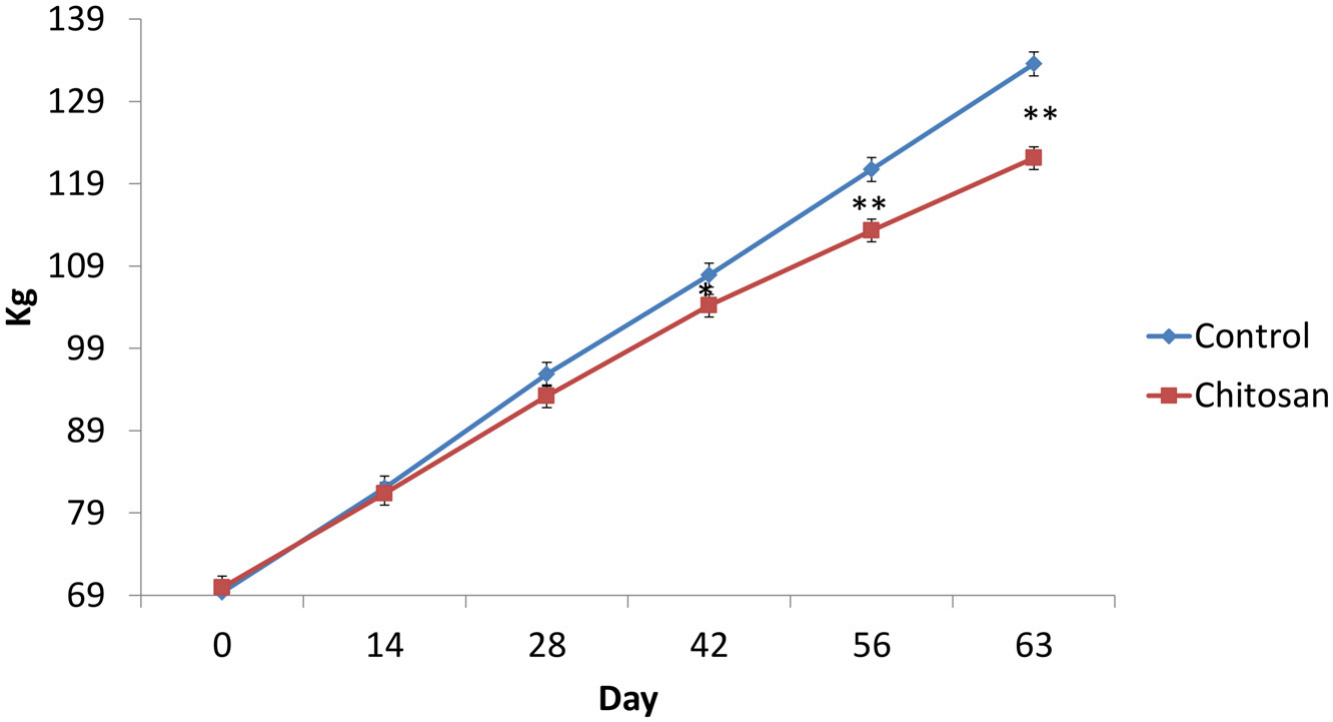
Effect of dietary supplementation on body weight over time at days 0, 14, 28, 42, 56 and 63. *P<0.05 **P<0.001 Treatment effect P< 0.001. Time effect P< 0.001. Time x treatment effect P< 0.01. Values are means, with their standard errors represented by vertical bars.

The effect of chitosan supplementation on body weight over time is presented in Fig 1. The effects of chitosan supplementation on pig performance and carcass characteristics are presented in Table 4. Pigs offered the chitosan diet had lower dietary intake (P< 0.01) and body weight gain (P< 0.05) during the experiment (days 0–63) (P< 0.01), and lower final body weight (P< 0.05) compared with pigs offered the basal diet. There was no effect of chitosan inclusion on feed conversion ratio (P>0.05). Animals offered chitosan had lower backfat depths and total carcass fat content compared to the basal group (P< 0.05). Lean meat percentage was higher in chitosan supplemented pigs (P<0.01) when compared to the basal group.

**Table 4:** Effect of dietary supplementation on growth performance and carcass characteristics (least-square means and SEM).

Performance	Control	Chitosan	SEM	P value
Dietary intake (kg/d)	2.99	2.67	0.05	0.001
Body weight gain (kg/d)	0.88	0.79	0.03	0.042
Feed efficiency ratio (kg/kg)^††^	3.57	3.30	0.37	0.593
**Carcass characteristics**				
Carcass weight (kg)	93.8	90.1	1.52	0.092
Kill-out proportion (%)	74.7	75.5	0.38	0.151
Back-fat depth (mm)	12.8	11.4	0.37	0.011
Fat content (kg)	36.4	33.6	0.74	0.012
Lean meat (%)	58.2	59.7	0.38	0.008
Loin eye muscle depth (mm)	55.3	57.5	1.39	0.258

SEM, standard error of mean.

^††^ Body weight gain/ dietary intake.

### Coefficient of apparent ileal digestibility and coefficient of total tract digestibility

Pigs offered the chitosan diet had decreased CAID of DM and GE (P< 0.05) compared with the control group (Table 4). Pigs offered the chitosan diet had reduced CATTD of GE and N compared to the control group (P< 0.05) (Table 5).

**Table 5:** Effect of dietary treatment on the coefficient of apparent ileal digestibility (CAID) and the coefficient of apparent total tract digestibility (CATTD) of dry matter (DM), nitrogen (N), ash, gross energy (GE) and crude oil (least square means and SEM).

	Control	Chitosan	SEM	P value
**CAID %**				
DM	79.05	73.01	1.800	0.035
N	73.77	64.01	3.637	0.084
Ash	37.02	35.51	8.709	0.906
GE	78.69	72.31	1.848	0.032
**CATTD %**				
DM	82.01	81.00	0.500	0.192
N	79.89	78.06	0.481	0.019
Ash	50.24	47.96	3.165	0.620
GE	81.30	80.25	0.354	0.050
Crude oil	78.86	77.29	1.649	0.515

SEM, standard error of the mean.

### Serum leptin

**Fig 2:**
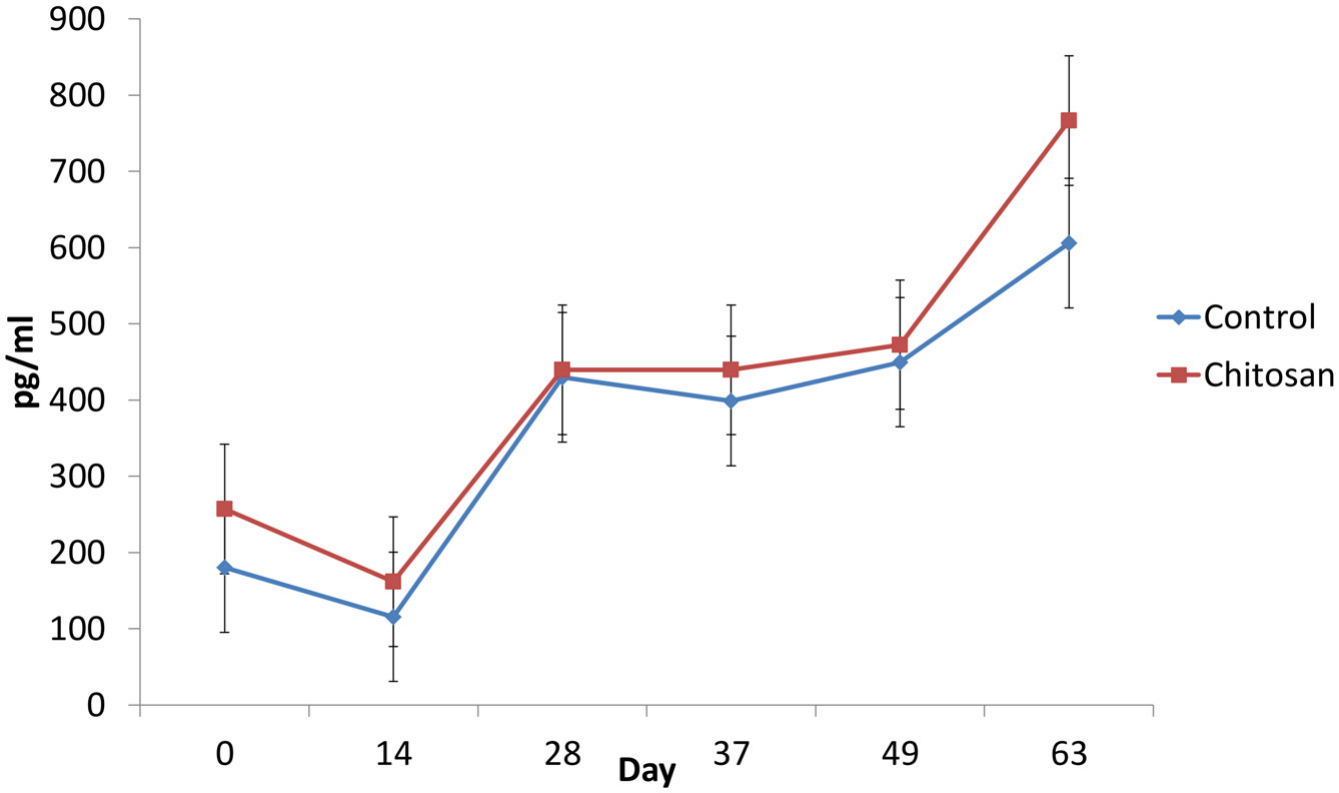
Effect of dietary supplementation on serum leptin levels over time at days 0, 14, 28, 37, 49 and 63. Treatment effect (P<0.05). Time effect (P<0.05). Values are means, with their standard errors represented by vertical bars.

There was a time effect (P<0.05) and treatment effect (P<0.05) on serum leptin concentrations. Serum leptin concentrations were higher in pigs offered the chitosan diet compared with the basal treatment group (P< 0.05) (Fig 2). There was no interaction between time and treatment on serum leptin concentrations (P> 0.05).

### Nutrient transporter gene expression

The effects of chitosan supplementation on nutrient transporter gene expression are presented in Table 6. The gene expression of *FABP2* was down-regulated in animals supplemented with chitosan compared with the basal group (Bonferroni test, P< 0.05). No supplementation effect was observed on the gene expression of the remaining nutrient transporters (P> 0.10).

**Table 6:** Effect of dietary treatment on nutrient transporter gene expression between treatments and in the duodenum, jejunum and ileum (Relative expressions) (least square means and SEM).

	Treatment			Region				Treatment	Region
*Gene*	Control	Chitosan	SEM	Duodenum	Jejunum	Ileum	SEM	P value *	P value *
*PEPT1*	1.171	1.059	0.2045	1.095	1.149	1.101	0.2496	0.6993	0.9851
*SGLT1*	1.694	1.072	0.2630	1.477	1.536	1.136	0.3223	0.1032	0.6275
*GLUT1*	0.952	0.705	0.1173	0.495	1.036	0.941	0.2553	0.1468	0.0465
*GLUT2*	1.406	1.012	0.2022	1.350	1.506	0.772	0.2474	0.1770	0.0896
*GLUT5*	1.046	1.179	0.2095	1.082	1.390	0.866	0.2566	0.6554	0.3393
*GLUT7*	0.996	1.470	0.2555	1.125	1.772	0.802	0.3133	0.1994	0.0865
*FABP2*	1.558	0.948	0.1954	1.240	1.970	0.550	0.2384	0.0336	0.0005
*CD36*	0.729	0.919	0.2094	0.495	1.036	0.941	0.3502	0.5237	0.3285

*PEPT*, peptide transporter; *SGLT*, sodium-glucose linked transporter; *GLUT*, glucose transporter; *FABP*, fatty acid-binding protein; *CD36*, cluster of differentiation 36; SEM, standard error of mean.

*No interaction between treatment and region P> 0.05.

### Digestive enzyme gene expression

There was no effect of dietary supplementation on digestive enzyme gene expression in the duodenum, jejunum and ileum (P> 0.10) (data not shown).

### Microbiology

The effects of chitosan supplementation on selected microbial populations in both the caecum and colon are presented in Table 7. Pigs offered chitosan had decreased GCN of *Lactobacillus* spp. in both the caecum (P< 0.05) and colon (P< 0.001), and increased GCN of *Bifidobacterium* in the caecum (P <0.05) compared to the control group. Animals offered chitosan had decreased GCN of *Firmicutes* in the colon compared with the control group (P< 0.05). There was no dietary supplementation effect on *Bacteroidetes* and *Enterobacteriaceae* population in either the caecum or colon (P> 0.10).

**Table 7:** Effect of dietary supplementation on selected microbial populations in the caecum and colon (GCN/g digesta) (least-square means and SEM).

	Control	Chitosan	SEM	P value
**Caecum**				
*Bacteriodes*	11.20	11.15	0.217	0.894
*Firmicutes*	10.95	10.87	0.111	0.639
*Bifidobacterium*	9.01	9.706	0.229	0.045
*Lactobacillus* spp.	11.41	10.79	0.205	0.047
*Enterobacteriaceae*	8.60	9.24	0.268	0.107
**Colon**				
*Bacteriodes*	11.16	10.83	0.247	0.361
*Firmicutes*	10.93	10.64	0.076	0.015
*Bifidobacterium*	9.49	9.89	0.181	0.136
*Lactobacillus* spp.	11.36	10.59	0.120	0.001
*Enterobacteriaceae*	8.17	8.70	0.297	0.229

SEM, standard error of the mean. Gene copy number/ g digesta.

### Volatile fatty acids concentration

The effect of chitosan on volatile fatty acid concentrations is shown in Table 8. Total VFA concentration was increased in the colon of chitosan-supplemented animals compared with the control group (P<0.05). Chitosan-supplemented animals had increased molar proportions of acetate in the colon compared to the control group (P<0.05).

**Table 8:** Effect of dietary supplementation on total volatile fatty acid (VFA) concentration and proportions in the colon (least-square means and SEM).

Molar proportions	Control	Chitosan	SEM	P value
Total VFA (mmol/g digesta)	104.8	114.8	3.44	0.050
**Molar Proportions**				
Acetic acid	65.92	71.58	1.982	0.050
Propionic acid	20.65	23.25	1.561	0.256
Isobutyric acid	1.41	0.76	0.328	0.183
Butyric acid	13.98	14.33	1.212	0.840
Isovalaeric acid	1.30	1.08	0.120	0.215
Valeric acid	1.77	1.95	0.328	0.714

VFA, volatile fatty acids; SEM, standard error of the mean.

## Discussion

The present study hypothesised that dietary supplementation of chitosan would cause a reduction in dietary intake, body weight and carcass fat content with a down-regulation of long chain fatty acid transporter and digestive enzyme gene expressions, while altering gut microbial populations in favour of a leaner body weight. The response observed in animals offered the chitosan supplement, such as reduction in dietary intake and body weight gain, down-regulation of *FABP2* gene expression, increase in serum leptin concentration, and significant alterations in gut microbial populations supports the hypothesis.

Dietary supplementation with chitosan reduced dietary intake and weight gain, similar to findings by Walsh *et al*. [2]. The reduction in weight gain may be attributable to a number of factors. Firstly, it may be due to the reduction in dietary intake observed in animals supplemented with chitosan with direct effects on weight gain. Secondly, the reduction in body weight gain may be due to the increases in serum leptin concentrations which subsequently affect appetite. The hormone leptin regulates body weight by controlling food intake and energy expenditure [5]. The ability of leptin to regulate appetite and energy expenditure in rodents with subsequent loss of adipose tissue has led to the description of leptin as an anti-obesity hormone [29]. Thirdly, the reduced weight gain may be attributable to decreased nutrient digestibility, evidenced by reductions in the CAID and the CATTD of GE. Furthermore, reduced energy digestibility and reduced weight gain was evident in the carcass composition. The overall lean meat percentage was higher in chitosan supplemented animals while carcass fat content and back fat depths were lower relative to the control group, indicating that chitosan supplemented animals had a lower and leaner body weight. Interestingly, while carcass fat content and fat depths measured were lower in chitosan supplemented animals, leptin levels were high, suggesting chitosan had a direct effect on leptin levels independent of body fat stores.

There is growing evidence that the small intestine can play an important role in the etiology of obesity, serving as a gatekeeper at the physical interphase between the body and the diet [30]. The small intestine is the primary site for absorption and assimilation of nutrients. In the present study chitosan supplementation had no effect on lipase and maltase gene expression in the small intestine, suggesting that chitosan may act only on specific cellular targets. Following nutrient breakdown by digestive enzymes, nutrients are then transported from the extracellular setting into the blood stream by nutrient transporters [11]. In the present study, *FABP2* gene expression was down-regulated in chitosan supplemented animals. Fatty acid binding protein 2 is responsible for the uptake of long chain fatty acids and is thought to help maintain energy homeostasis by functioning as a lipid sensor [31]. The down-regulation of *FABP2* may have reduced the absorption of lipids, thus leading to the observed reduction in body mass. Although not significant, a numerical decrease in the CATTD of ether extract was associated with chitosan supplemented animals. Unfortunately, due to a lack of availability of digesta, the CAID of crude oil was not measured.

Accumulating evidence indicates that intestinal microbiota play a significant role in the development of obesity [32, 33]. Differences in microbiota composition in the gut have been observed between lean and obese individuals [8, 34]. The bacterial phylum *Firmicutes* has been extensively researched for its role in obesity, with studies observing an increase in *Firmicutes* populations in the caecum and colon of obese individuals [33, 35]. In the present study, the GCN of *Firmicutes* was reduced in the colon of chitosan-supplemented animals. Although the influence of *Firmicutes* on obesity is still unclear, it has been suggested that *Firmicutes* promote adiposity [36]. *Firmicutes* metabolise food substrates more completely [37] thereby enabling more efficient absorption of calories and subsequent weight gain. In the present study, the reduction in GCN of *Firmicutes* in the colon of chitosan-supplemented animals may partially explain the reduction in body weight gain.

It has also been reported that *Lactobacillus* spp. [38] and *Bifidobacterium* spp. may have a role in weight regulation [39]. The intestinal microbiota has an important role in the fermentation of non-digestible carbohydrates into short chain fatty acids. *Bifidobacterium* and *Lactobacillus* are known to ferment carbohydrates to acids; *Lactobacillus* spp. produce lactate, and *Bifidobacterium* spp. predominantly produce lactate and acetate. In the present study, GCN of *Lactobacillus* spp. was decreased in the caecum and colon and GCN of *Bifidobacterium* spp. was increased in the caecum of animals supplemented with chitosan. Chitosan supplementation also resulted in an increased production of acetic acid. Acetate is the main VFA produced from the fermentation of non-digestible carbohydrates [40]. Additionally, the total VFA concentration was increased in the colon of animals supplemented with chitosan. Chitosan supplemented animals had lower CAID of DM and GE compared to the control group suggesting there may be more material available for fermentation in the colon of chitosan supplemented animals. An increased VFA concentration has been shown to indicate increased microbial activity in the gastrointestinal tract [41]. Furthermore, VFAs have been suggested to regulate insulin and glucagon secretion [40]. Glucagon and insulin are part of a feedback system that regulates blood glucose, aiding in the control of appetite. Insulin is positively correlated with long-term energy balance [42]. In a study using mice fed a high fat diet, with the objective to induce obesity and diabetes, it was observed that fat mass was reduced when the diet was supplemented with a *Bifidobacterium* strain, leading to decreased body weight gain and improved glucose tolerance [43]. Interestingly, the present study had similar effects whereby chitosan supplemented animals had significantly reduced weight gain and increased *Bifidobacterium* abundance in the caecum when compared to the control group.

*Lactobacillus spp*. and *Bifidobacterium* spp. also function as potent probiotic agents [44]. Chitosan appears to exhibit prebiotic properties by increasing the population of *Bifidobacterium* spp. in the caecum, thus having a positive effect on gut health. However, GCN of *Lactobacillus* spp. was decreased in the caecum and colon of chitosan-supplemented animals. Although *Lactobacillus* is considered a probiotic agent, recently, it has been suggested that probiotics, and in particular *Lactobacillus* are contributing to human obesity [39]. Interestingly, gut microbiota associated with obesity have been found to be enriched in *Lactobacillus* and depleted in *Bifidobacterium* similar to the findings in the present experiment within the control group of animals, while chitosan supplemented animals had a decrease in *Lactobacillus* and an increase in *Bifidobacterium* populations.

In conclusion, the present study demonstrated that dietary supplementation of prawn derived chitosan reduces feed intake and body weight in a pig model. This effect may be orchestrated through multiple responses both within the intestinal tract and bloodstream including; decreased nutrient digestibility, decreased FA transporter gene expression, increased serum leptin and altered gut microflora.
